# Mixed-species groups in bats: non-random roost associations and roost selection in neotropical understory bats

**DOI:** 10.1186/s12983-021-00437-6

**Published:** 2021-10-12

**Authors:** Detlev H. Kelm, Ulf Toelch, Mirkka M. Jones

**Affiliations:** 1 Zoology 2, University of Erlangen-Nuremberg, Erlangen, Germany; 2 Leibniz-Institute for Zoo and Wildlife Research, Berlin, Germany; 3 Estación Biológica de Doñana, Seville, Spain; 4 Berlin Institute of Health at Charité – Universitätsmedizin Berlin , QUEST Center for Responsible Research , Berlin, Germany; 5 Institute of Biotechnology, HiLIFE Helsinki Institute for Life Science, University of Helsinki, Helsinki, Finland

**Keywords:** Artificial bat roosts, *Carollia*, Communal roosting, *Glossophaga*, Heterospecific animal groups, Interspecific interaction, Mixed-species groups, Roosting ecology, Species association

## Abstract

**Background:**

Mixed-species groups in animals have been shown to confer antipredator, foraging and other benefits to their members that may provide selective advantages. In most cases, however, it is unclear whether functional benefits are a principal driver of heterospecific groups, or whether groups simply result from simultaneous exploitation of common resources. Mixed-species groups that form independently of environmental conditions may, however, evidence direct benefits of species associations. Bats are among the most gregarious mammals, with sometimes thousands of individuals of various species roosting communally. Despite numerous potential functional benefits of such mixed-species roosting groups, interspecific attraction has never been shown. To explore alternative explanations for mixed-species roosting, we studied roost selection in a speciose neotropical understory bat community in lowland rainforest in Costa Rica. Long term roost data were recorded over 10 years in a total of 133 roosts comprising both natural roosts and structurally uniform artificial roosts. We modelled bat roost occupancy and abundance in each roost type and in forest and pasture habitats to quantify the effects of roost- and environmental variability.

**Results:**

We found that bat species presence in natural roosts is predictable from habitat and structural roost parameters, but that the presence and abundance of other bat species further modifies roost choice. One third of the 12 study species were found to actively associate with selected other bat species in roosts (e.g. *Glossophaga commissarisi* with *Carollia sowelli*). Other species did not engage in communal roosting, which in some cases indicates a role for negative interspecific interactions, such as roost competition.

**Conclusions:**

Mixed-species roosting may provide thermoregulatory benefits, reduce intraspecific competition and promote interspecific information transfer, and hence some heterospecific associations may be selected for in bats. Overall, our study contributes to an improved understanding of the array of factors that shape diverse tropical bat communities and drive the dynamics of heterospecific grouping in mammals more generally.

**Supplementary Information:**

The online version contains supplementary material available at 10.1186/s12983-021-00437-6.

## Background

Animals often assemble in mixed-species groups comprising two or more species, with one to thousands of individuals of one species occurring in relative proximity to individuals of other species [1, 2]. The characteristics of these groups can vary considerably. Some interspecific associations are passive, with little direct interaction between species, such as in most mixed-species ungulate herds [3]. In other mixed-species groups there may be coordinated behavioural interactions between species, as has been found in primates [4, 5]. Similarly, the stability of mixed-species groups may vary from short periods of hours to almost permanent associations [1]. Mixed-species groups are relatively common and well-studied in birds and fish [6, 7]. In mammals, this phenomenon has received less attention and the best examples are mainly from primates [1, 2].

The formation of mixed-species groups has commonly been explained by benefits to at least one member species. The main hypothesized benefits are similar to those for the formation of single species groups, i.e., increased predator vigilance, deterrence and evasion, and increased foraging efficiency [1, 2]. For example, when individuals of one species join a group of another species with which they share common predators, they may benefit from both protection within a larger group and increased vigilance provided by heterospecific group members. In some cases, heterospecific vigilance may even complement each species’ own predator detection capability [2, 8]. This may allow time to be re-allocated from vigilance to foraging, thereby increasing foraging rate [9, 10]. Furthermore, when the group’s member species differ in resource utilization, the advantages of the association will not be diminished through increased competition, unlike in monospecific groups. Therefore mixed-species groups may have significant positive impacts on the survival and fitness of their member species and perhaps significantly shape their ecological niches [2, 11, 12].

Heterospecific aggregations may, however, also arise due to the simultaneous exploitation of a common resource, due to other environmental factors, or simply by chance. Even when significant benefits of an interspecific association seem likely, it may be unclear whether there is selection for this behaviour if, for example, a shared dependence on environmental variables cannot be ruled out. Indeed, in the majority of cases, especially in mammals, it remains unclear whether there is actually selection for mixed-species groups arising from such functional benefits. This may be because (1) behavioural observations to test the proposed functional benefits are lacking or (2) the association’s net impact on fitness, after accounting for negative effects, such as competition, is unclear [13].

Communally roosting bats are among the most gregarious mammals. Diurnal roosts, i.e. places to shelter from predation and adverse climate and to rest during daylight hours, may host several up to millions of individuals. They are mostly located in trees or rock crevices and caves and are an essential resource for bats [14]. Extreme gregariousness in many bat species can be explained by various benefits that accrue from roosting in groups, most of which are valid for animal aggregations in general, such as predator deterrence, the dilution effect and increased vigilance [15–17]. Bats may also gain energetic benefits from roosting in groups, because the costs of thermoregulation are reduced either by clustering or simply by increasing ambient roost temperature through body heat [18, 19]. Additionally, communal roosting allows for information transfer, e.g. on food sources or other roosts [20, 21].

Many bat communities are speciose, especially in the tropics. For example, Rex et al. [22] estimated up to 100 bat species to occur sympatrically in a neotropical rainforest. Many bat species use similar types of roosts and interspecific roost associations are common. Within these, heterospecific individuals often roost in close physical proximity [14, 23]. In fact, heterospecific roosting groups in bats may be the most frequent mixed-species groups in mammals. We argue that the functional benefits that apply to aggregations of bats in monospecific groups may in some cases be similarly or even more beneficial for members of mixed-species roosting groups.

Diurnal roosts are, however, likely to represent a limited resource for bats, and their availability clearly influences species’ distributions [24]. Indeed, it has been generally assumed that bat species aggregations in roosts simply result from the simultaneous use of a common, potentially limited resource. This may also result in roost competition between species with similar roost requirements. Behavioural studies on bats in mixed-species roost associations are very rare, despite the potential for both interspecific conflicts and mutual benefits [25]. Some species pairs have been found to co-occur in roosts more frequently than expected by chance, and active species associations have been proposed to explain these [26, 27]. Nonetheless, no study has convincingly shown an active association of bat species in roosts that is independent of environmental variables. For example, Arita and Vargas [28] rejected the hypothesis of an active association of bat species in cave roosts in Mexico. Interspecific social bonding [29] and information transfer between bats sharing roosts has only recently been confirmed [30], indicating the potential for further functional benefits of mixed-species roost associations. Similarly, besides some indications of aggressive interspecific exclusion from roosts [31], information on roost competition and its influence on species distributions in bats is scarce [32]. Detailed information on species-specific roost selection is also often lacking, especially in speciose tropical communities.

The aims of this study were to (1) analyse mixed-species roosting groups in a species rich neotropical bat community and identify any consistent interspecific roost associations, (2) assess whether competition is an important factor determining roost selection in bats, and (3) describe how roost characteristics, including physical dimensions and temperature, and interspecific interactions interact to define species-specific roost selection. We predicted that active positive associations should arise between species that (a) have a low dietary niche overlap, (b) have complementary thermoregulatory strategies, and (c) share common predators and complement each other in vigilance and predator deterrence. We also predicted that roost competition should arise in ecologically similar species, i.e. those with a common diet, and between species with incompatible roosting behaviours.

## Results

Natural roosts were observed 543 times and in total 11 bat species were encountered. On 23 occasions bats were present but could not be identified to the species level. Structurally similar artificial roosts in the forest and in the disturbed habitat were observed 593 and 732 times respectively, and in total nine bat species were encountered over both habitats. In 8 and 74 cases in the forest and disturbed habitat, respectively, bats were present, but could not be identified to the species level (Additional file 1: Table S1). *Trachops cirrhosus* (T.cir), *Micronycteris hirsuta* (M.hir), *Hylonycteris underwoodi* (H.und) and *Desmodus rotundus* (D.rot) were never found in the disturbed habitat, whereas *Glossophaga soricina* (G.sor) was never recorded in any roost in the forest habitat. In 27% of all observations, roosts were unoccupied. This proportion did not differ between the three datasets (χ^2^ = 1.52, df = 2, *P* = 0.47).

All natural roosts were tree roosts, most often in hollow standing tree trunks (54% of all roosts) and hollow fallen tree trunks (38%), while others were in open cavities of split trees and buttresses (8%). Of those roosts in or on standing trees (*n* = 55) 76% were in three tree species (*Dipteryx panamensis*: *n* = 17, *Terminalia oblonga*: *n* = 13, *Pentaclethra macroloba*: *n* = 12, *Vitex cooperi*: *n* = 2, *Virola koshnyi*, *Ochroma pyramidale*, *Lecithes ampla*, *Hura crepitans*, *Hieronyma alchorneoides*, *Apeiba membranacea*: each *n* = 1, unidentified: *n* = 5).

### Bat species roost associations

**Fig. 1:**
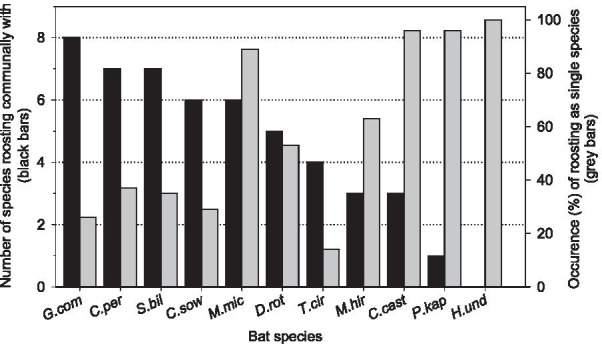
Number of bat species a given species was found to share a roost with at least once (black bars; not necessarily simultaneously) and proportion of observations (%) of monospecific roosting (grey bars) in natural roosts. For species abbreviations see text and Additional file 1: Table S1

Two or more species were recorded simultaneously in 25% of all observations of bats in natural roosts (*n* = 377 observations) with a maximum of five species simultaneously. The corresponding figures for occupied artificial roosts in the forest and disturbed habitat, respectively, were 41% (*n* = 427) and 55% (*n* = 445) of observations, respectively, with a maximum of three species roosting simultaneously. Some species used a variety of natural roost types that were also used by other species while others rarely or never shared roosts with other species. For example, *Carollia sowelli* (C.sow) and *Glossophaga commissarisi* (G.com) used roosts that were often colonized by other species and frequently roosted communally, while *Peropteryx kappleri* (P.kap) and *Hylonycteris underwoodi* usually selected roosts that were not used by other species. *Saccopteryx bilineata* (S.bil) usually roosts on tree trunks. However, in some cases this species was also found roosting inside large hollow trees and in tree cavities. *Micronycteris microtis* (M.mic) and *C. castanea* (C.cast) were found to roost with conspecifics alone in 89% and 96% of all observations (*n* = 55 and 80 observations, respectively) although these species were frequently found in roosts that were also used by other species (Fig. 1).

**Fig. 2:**
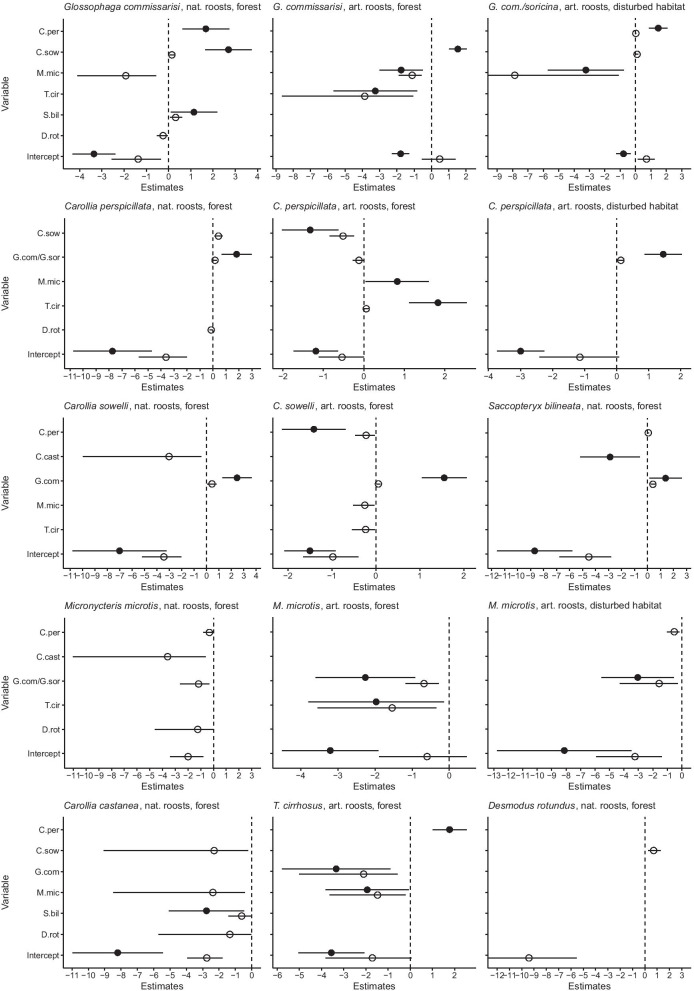
Results of GLMM on the effect of the presence (black circles) and abundance (open circles) of bat species on the incidence of given bats species in natural and artificial roosts in the forest habitat and artificial roosts in the disturbed habitat. The plots only show those species for which a significant effect was detected. Models without any significant effects are not presented. For species abbreviations see the text and Additional file 1: table S1. The error bars are 95% credible intervals

Some species co-occurred in roosts more often than expected by chance. The clearest species associations were those of the small nectarivorous species *G. commissarisi* and *G. soricina* with either one of the larger frugivorous species *C. perspicillata* (C.per) or *C. sowelli*. Both the presence/absence and abundance models for artificial and natural roosts generally suggest significant positive associations between these species (Fig. 2). In 66% of all observations of *G. commissarisi* in natural roosts (*n* = 65) and 80% of all observations in artificial roosts in the forest (*n* = 122) this species co-occurred either with *C. sowelli* or *C. perspicillata* and there was often close physical contact between heterospecific individuals. In 87% of all observations of *G. commissarisi* and *G. soricina* in artificial roosts in the disturbed habitat (*n* = 263) either *C. sowelli* or *C. perspicillata* was present. Whilst in the disturbed habitat *G. commissarisi* and / or *G. soricina* were most frequently associated with *C. perspicillata*, in artificial roosts in the forest the association of *C. sowelli* and *G. commissarisi* was dominant. Only the models for *G. commissarisi* in artificial roosts in the forest did not show a significant support for an association with *C. perspicillata*. Models of the association of the insectivore/carnivore *Trachops cirrhosus* and C. *perspicillata* showed positive effects of the presence of one species on the occurrence of the other in artificial roosts in the forest habitat. The models also showed positive associations of *G. commissarisi* and *C. perspicillata* with the insectivorous *Saccopteryx bilineata* in natural roosts.

Examples of negative associations were also identified. In natural and artificial roosts in the forest, the incidence of *M. microtis* was negatively affected by the presence and abundance of several other species in natural and artificial roosts including *G. commissarisi* and *T. cirrhosus*. *Carollia castanea* was also negatively associated with various other species in natural roosts. Similarly, *C. perspicillata* and *C. sowelli* were negatively associated in artificial roosts in the forest (Fig. 2). Models with pooled data for the sibling species *C. perspicillata* and *C. sowelli* show similar results (Additional file 1: Fig. S1).

**Fig. 3:**
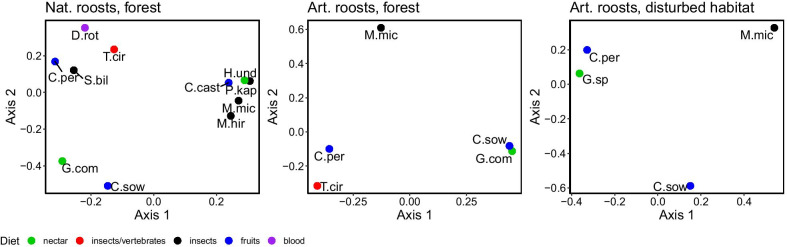
Principal Coordinate Analysis of species occurrences in natural and artificial roosts in the forest and artificial roosts in the disturbed habitat. For species abbreviations see the text and Additional file 1: table S1

The ordination of bat associations in roosts supports the GLMM results, with *G. commissarisi* being associated with *C. sowelli* in the forest and with *C. perspicillata* in the disturbed habitat (Fig. 3). For natural roosts, the ordination also groups those species together that use similar roost types, such as species that occupy roosts in hollow fallen trees (e.g. *C. castanea, H. underwoodi, M. microtis, P. kappleri*) and species that typically occupy large standing hollow trees (*C. perspicillata*, *D. rotundus*, *S. bilineata*, *T. cirrhosus*).

### Species-specific selection of natural roosts

**Fig. 4:**
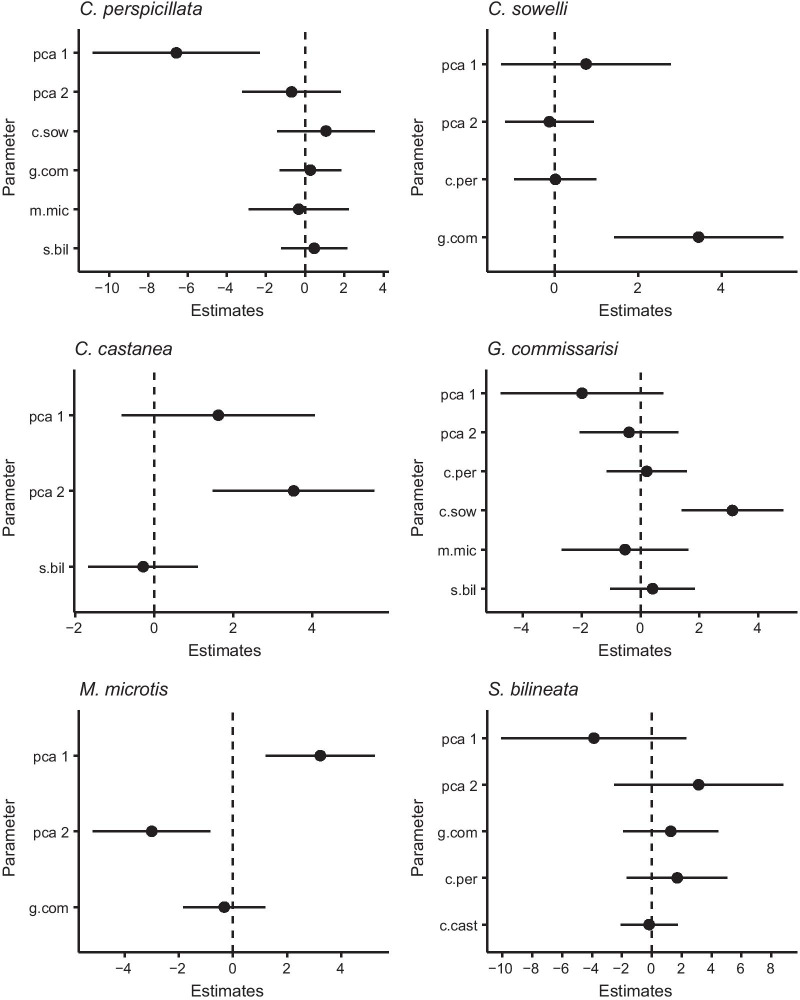
Results of GLMs predicting the presence/absence of six bat species as a function of roost variables (axes 1 and 2 of a PCA summarizing seven roost parameters, Table 1) and the presence/absence of the other bat species

Regression models analysing the influence of roost parameters on roost selection show species-specific patterns of selectivity (Fig. 4, Additional file 1: Table S2). Some species preferentially roost closer to the ground, e.g. in fallen trees (*M. microtis, C. castanea, H. underwoodi*), while others select roost sites higher above ground, e.g. in hollow trunks of standing trees (*C. sowelli, C. perspicillata*, *G. commissarisi*). Some species (e.g. *T. cirrhosus*, *D. rotundus*) were exclusively found in the largest tree cavities with inner diameters exceeding 1 m and an inner height of 11 ± 7 m (n = 11). For *M. microtis* and *C. castanea*, physical roost characteristics were the factors most strongly influencing their roost selection. In contrast, for *G. commissaris*i and *C. sowelli* the presence of other bat species was more important in determining their incidence in roosts, via a positive association. We used the first two axes of the PCA of roost parameters as a proxy for these variables. Table 1 shows the correlation of the original roost variables with PC axes 1 and 2. The environmental variables with the highest loadings on PC 1 and 2 were the distance from the roosting site to the ground and to the roost entrance, respectively. In an analysis of species richness by roost type, we found that the largest roosts in standing trees were used by most species and that the inner height of roosts was a significant predictor of the number of species using a roost (Additional file 1: Fig. S2).

**Table 1:** Correlations of seven roost parameters with the first two axes of a principal component analysis (PCA) summarizing these variables

	Tree type (standing vs. fallen)	Roost volume	Size of the entrance	Vegetation density	Canopy height	Distance roosting site to entrance	Roosting site above ground
PC1	− 0.81	− 0.66	− 0.56	0.29	− 0.71	− 0.39	− 0.89
PC2	0.44	− 0.59	0.25	− 0.28	− 0.09	− 0.81	0.22

We found that temperatures in more exposed roosts followed daily ambient temperature dynamics with levelled out peaks, and that daily roost temperature fluctuations decreased with the size and height of roosts. Roosts within large hollow trunks of standing trees had more stable daily mean temperatures, averaging 25 ± 1 °C, and with a temperature gradient of on average 4 °C towards the entrance, which was usually at the bottom of the roost between the tree’s buttresses (Additional file 1: Fig. S3). Temperatures in artificial roosts in the forest were most similar to those of medium sized roosts in standing trees, while temperatures in artificial roosts in the disturbed habitat were less stable, but nonetheless buffered ambient temperature extremes.

## Discussion

### Mixed-species groups

We show that mixed-species roosting groups of Neotropical bats are not random species aggregations that simply occur due to the exploitation of a common resource, the bats’ diurnal roosts. The composition of roosting groups is influenced by both species-specific preferences for particular roost types and by interspecific attraction and repulsion. By constructing uniform artificial roosts, we were able to minimize the influence of variation in structural roost characteristics on roost choice. In these roosts, there was clear evidence of the impact of interspecific interactions on roost choice. In natural roosts, we also found evidence of species-specific roost-type preferences.

Interspecific attraction in roosts between some species pairs suggests that there may even be selection for these species associations. The benefits of mixed-species roosting may outweigh its costs and even provide higher overall benefits compared to roosting in monospecific groups. The strong propensity of most bat species to aggregate in roosting groups suggests a positive cost-benefit ratio of communal roosting. These benefits may include protection against predation through detection- and dilution effects [33] and thermoregulatory benefits [18, 19]. The principal costs of aggregation result from increased resource competition, conspicuousness to predators and propagation of diseases and parasites [25, 34, 35]. Mixed-species groups may provide the benefits of aggregation, but with reduced concomitant costs, e.g. from competition, compared to similar-sized monospecific groups. Therefore, we predicted positive associations between species with different diets, due to the lack of foraging competition within such associations. Indeed, the most frequent positive species association was that between the frugivorous *C. perspicillata* or *C. sowelli* (18–20 g) and the smaller-bodied mostly nectarivorous *Glossophaga commissarisi* (8–10 g). Although *Glossophaga*-species have been found to seasonally enrich their nectar diet with fruits also consumed by *Carollia* [36–38], this temporary dietary overlap may not represent an entirely negative cost of association, but could be beneficial if there is interspecific information transfer in roosts on the location of foraging sites, via olfactory cues. Such social learning in roosts related to foraging has been shown for frugivorous *C. perspicillata* [21] and *Uroderma bilobatum* [30]. The information centre hypothesis [39] describes information transfer at roosts in birds, and heterospecific information has been argued to be a driving factor influencing the formation and maintenance of mixed-species groups in other animals [40].

We also predicted that positively associated species might share common thermoregulatory strategies. In support of this prediction, we regularly observed interspecific clustering between individuals of *Carollia* and *Glossophaga*. This association between the two genera has been described previously [26]. Particularly for the smaller bodied *Glossophaga*-species, thermoregulatory benefits of clustering with the larger *Carollia*-species are likely. Both genera have similar thermoregulatory strategies, including diurnal torpor [41, 42], and conspecific clustering has been shown to reduce thermoregulatory costs in *Glossophaga* and other tropical bat species [18, 43].

The literature on predation for the bat species encountered in our study is scarce, but despite size differences between study species, predators attacking bats in roosts may not be selective. Therefore, besides protection by the dilution effect, active predator deterrence may be important in mixed-species roosts [16]. Mixed-species groups may also be more effective at defending roosts against common roost competitors, and diseases and parasites may be less prevalent in mixed-species groups compared to similar sized monospecific groups, since many bat parasites are host-specific [44, 35]. For single males of species that form harem groups, such as *Carollia* and *Glossophaga*, roosting with heterospecifics may be particularly attractive, as this would prevent inter-male competition, and because mixed-species groups may nevertheless attract conspecific females. For other roost associations, such as *C. perspicillata* with the insectivorous/carnivorous *T. cirrhosus*, the benefits of mixed-species roosting would be similar to those mentioned above. Social network analyses of heterospecific roost groups in bats support the hypothesis that mixed-species bat groups provide fitness benefits. For example, Ancillotto et al. [29] presented evidence for interspecific inter-individual bonding between communally roosting bat species, a phenomenon that could facilitate the evolution of interspecific attraction and association in roosts as well as inter-species information transfer, which has been argued to be key for the formation of mixed-species groups [40].

### Roost competition

We found the strongest evidence for roost competition in the smallest study species, *M. microtis*, with an average body weight of just 5 g. We observed that this species was often the first to colonize new artificial roosts, but our association models showed that the incidence of *M. microtis* in artificial roosts was negatively affected by the presence of other species. This was evidenced by the eventual replacement of *M. microtis* in artificial roosts by other species. While artificial roosts simulate well-protected roosts in hollow trunks of standing trees, natural roosts of *M. microtis* were close to the ground in hollow logs of fallen trees that are most prone to predation and that were rarely used by other species. We hypothesize that rapid colonization of new artificial roosts by *M. microtis* may reflect strong exploratory behaviour necessitated by its inferior competitiveness for better protected roosts. By eavesdropping on *M. microtis*, other bat species might even be able to gain information on the availability of new roosts [45].

Our models indicated that *C. perspicillata* shared artificial roosts in the forest with *C. sowelli* less often than expected, which could arise due to competition and the strong dietary overlap in these two similar-sized species [37]. However, in artificial roosts in the disturbed habitat, there was no detectable effect of the presence or abundance of one species on the incidence of the other and in natural roosts *C. sowelli* conversely had a small positive effect on the incidence of *C. perspicillata*. These observations may be explained by similarities in their species-specific roost preferences in natural roosts on the one hand, and by the lower incidence of *C. sowelli* in the disturbed habitat on the other.

In both the presence-absence and abundance models of species association, the effects of the predictors generally had the same sign, although in some cases predictors had a significant effect in only one model type. However, we assume that the cost-benefit ratio of species associations will depend on the abundance ratio of the member species in mixed roosting groups, because antagonistic interactions and their related costs (e.g. energetic expenditure) may increase with group size, and species may respond differently to such costs. Comparisons between presence-absence and abundance model results may therefore help to identify such abundance-related costs of species associations.

Despite a high colonization rate in artificial roosts, roosts were unoccupied in 27% of observations, independently of roost type and site. Frequent roost switching, a common observation in bats, has been described by fission-fusion models [46] and may be triggered by or be a pre-emptive behaviour against predation events or parasite infestation of roosts [34]. As roost switching requires constant access to several roosts, the actual number of roosts that are available without inter- or intraspecific roost competition may be limited, despite the apparently constant availability of empty roosts. This assumption is supported by the fast and complete colonization of artificial roosts [37]. Inferences about roost availability based on snap-shot inventories of active and potentially available roosts, ignoring bat territoriality and intra- and interspecific roost competition for both preferred and secondary roosts, can hence result in erroneous conclusions on roost availability.

### Roost selection

The roost selection models showed that in some species roost characteristics were more important than interspecific interactions in determining roost selection, while for other species roost selection was primarily dependent on the presence of other bat species in roosts. The observed species-specific preferences for particular roost characteristics conformed with previous descriptions [47]. The lack of roost types other than tree roosts in our study was due to the absence of alternative roosting substrates (e.g. rock caves) in the area. Species that did not or only rarely colonize artificial roosts generally selected natural roosts that were very different from the artificial roosts, such as exposed roosts on tree trunks (*S. bilineata*) or roosts within or under fallen trees (*C. castanea*).

In natural roosts, interspecific behaviour may influence roost selection differently to in artificial roosts. While in artificial roosts bats roosted on the same plane on a surface of less than 1 m^2^, due to their flat roof design, more spacious natural roosts may provide a larger diversity of roosting sites and more space for avoiding antagonistic encounters between different bat groups. In our study, the inner height of roosts was a significant predictor of the maximum number of bat species using a roost. We interpret the positive roost association of *S. bilineata* with *G. commissarisi* and *C. perspicillata* in natural roosts to result from similar roost type preferences rather than a direct attraction between species. In fact, although *S. bilineata* may use cavity roosts, this species commonly roosts on the vertical surface of the trunks of large trees with a territorial behaviour that precludes communal roosting in smaller roosts [48].

The temperature gradient within large roosts illustrates the thermal diversity of roosts. It is even possible that specific roosting sites within roosts are selected based on species- or individual-specific thermoregulatory requirements, as small ambient temperature variations may significantly influence thermoregulatory behaviour and energetic expenditure in bats [42]. We did not, however, include roost temperature as a predictor in our roost selection models, because we assume roost selection for thermoregulatory reasons to be a secondary criterion at our study site, as ambient temperatures always remained stable within a physiologically non-critical range year round with daily fluctuations between c. 20–30 °C. Also, assessment of the role of temperature variation within and between roosts on roost selection would require continuous monitoring of the bats’ roosting behaviour in relation to roost temperature. In temperate regions, in contrast, where ambient temperatures reach well below the animals’ thermoneutrality, roost temperature has been found to be a principal factor determining roost selection in bats [49, 50].

We acknowledge that environmental variables at larger scales may also have influenced roost selection. The observation of a lower species richness in artificial roosts in the disturbed habitat than in the forest match previous findings of negative impacts of land use change on bat species occurrences in the tropics, especially arising from the conversion of forest to pasture [51, 52].

## Conclusions

Our study showed evidence of interspecific attraction in mixed-species roosting groups of bats in both natural and artificial roosts. In the latter, we propose that these associations are largely independent of roost characteristics. This suggests that there are evolutionary benefits of heterospecific roosting associations in bats, and that the benefits of heterospecific roosting may even outweigh those of roosting in monospecific groups. Species associations add additional dimensions to the realized niches of their member species [11] with implications for the evolution and coexistence of bat species in highly diverse tropical bat communities. Considering the substantial ecological impact of bats on their environment, e.g. as seed-dispersers [37, 53–55], an improved understanding of the evolution, structure and dynamics of bat communities is of particular importance.

## Methods

### Fieldwork

The study was carried out in two habitats, in tropical rainforest at La Selva Biological Station of the Organization for Tropical Studies (forest habitat) and in nearby pasture-forest mosaic landscapes (disturbed habitat) in the Caribbean lowlands of Costa Rica (10°26′ N, 83°59′ W) between 2000 and 2009. Regional bat species diversity is high, with a sympatric occurrence of 71 species [56].

We monitored bat species abundance in 89 natural roosts in the forest and in 44 artificial roosts [37] of which 21 were in the forest and 23 in the disturbed habitat. The natural diurnal roosts included were tree cavities within 10 m of the ground used by the understory bat community, mainly from the sub-family *Phyllostomidae*. Natural roosts were located by systematically searching tree trunks for roosts and by tracking bats that were radio-tagged during other studies. The artificial roosts were boxes made of sawdust concrete (0.54 or 0.74 m wide and 1.54 or 1.94 m tall) that were installed on the ground [37]. We did not include foliage and termite nest roosts. We did not inventory natural roosts in the disturbed habitat because roost abundance there is very low [37] and, unlike in the forest habitat, anthropogenic variables may influence roost choice.

Roosts were monitored repeatedly with a flashlight or an infrared video camera and we recorded individual numbers per bat species (group sizes > 20 were estimated), and their roosting site within the roosts. In some cases, we identified bats after capture with mist nets (70 D/2, mesh size 16 mm, Vohwinkel, Velbert, Germany) during evening exit from the roost.

For a subset of 58 natural roosts used by 10 bat species, we recorded 23 variables that described the roosts’ physical characteristics and close surroundings, of which we used seven in further analyses (tree type (standing vs. fallen), roost volume, size of the entrance, vegetation density within a 5 m radius of the roost entrance on a scale of 0–10, canopy height, distance of the roosting site from the entrance and above the ground). The size measures were taken with a laser range finder (Disto, Leica, Germany) or with a tape measure. For 48 roosts we also measured temperature at the roosting site and at different heights inside the roost as well as outside the roost with dataloggers (I-Button, Dallas Semiconductor, USA and HOBO Pro RH/Temp, HOBO Temp, Onset, USA). For comparison, ambient temperature data were acquired from the La Selva meteorological database.

### Data analysis – roost associations

To investigate patterns of interspecific species association in roosts, we analysed three separate datasets: (1) natural roosts, (2) artificial roosts in the forest habitat, and (3) artificial roosts in the disturbed habitat. The data were divided in this way because some species either did not occur in both habitats or did not occupy artificial roosts. We only included observations recorded at least seven days apart. This interval was chosen based on the observed dynamics of roost occupation to reduce temporal autocorrelation in the roost occupancy observations. There was a high probability of altered roost occupation after a week or longer and roost switching is a common phenomenon in bats [34, 46]. In the artificial roost dataset for the disturbed habitat, observations of the sibling species, *Glossophaga soricina* and *G. commissarisi* were pooled, because visual species identification was unreliable. Similarly, in some cases of very large roost aggregations of *Carollia perspicillata* and/or *C. sowelli* we either relied on capture data for species identification or, if this was not possible, the observations were pooled. Other observations of bats that could not be identified to species level were excluded from the analyses of species associations.

We fitted Bayesian Linear Mixed-Effects Models of bat species occupancy in roosts as a function of the occurrence of other species, defined as fixed variables (“presence-absence models”). Model fitting was with a binomial error structure and a normal prior using the “blme” package v. 1.0-4 [57] in R v. 3.5.1 [58]. To account for repeated measures, roost identity was included as a random term. We then tested whether the abundance of species in roosts was influenced by the abundances of other species. These Bayesian generalized linear mixed models (GLMM, “abundance models”) were fitted for each species with the R-package “brms”, v. 2.16.1 [59] with the abundances of other species as fixed variables and roost identity as a random effect on the intercept, with a negative binomial error distribution and loglink function. We also modelled potential zero inflation, as the datasets included a large number of zero observations. We used Cauchy priors for predictor variables to regularise estimates [60].

To visualize species associations in roosts, we applied principal coordinate analysis (PCoA) ordination [61] based on Jaccard’s similarity index calculated for each possible species pair based on their occurrences in the roosts using the R-package “vegan”, v. 2.5.2 [62]. This quantifies the number of roosts used by both species simultaneously divided by the total number of roosts used by one or the other or both species.

### Data analysis – roost parameters

To characterize the influence of roost characteristics on species-specific roost selection, we first ran a principal component analysis (PCA) on the seven selected roost variables to reduce the data variation into a few main axes and avoid collinearity amongst predictors using the “vegan” package [62] in R. We log-transformed those variables with highly skewed data (volume, size of entrance, distance of roosting site to entrance and ground) to reduce the impact of extreme values on the PCA. Then we fitted generalised linear logistic regression models with bat species occupancy as the response variable for those species observed in at least 10 roosts, i.e. *Saccopteryx bilineata*, *Micronycteris microtis*, *G.commissarisi*, *Carollia castanea*, *C. perspicillata* and *C. sowelli*. We included as predictors the first two PCs summarizing roost characteristics, and the presence/absence of those bat species that were found to significantly influence roost occupancy in the target species in the species association analyses. We applied multimodel inference to assess the statistical importance of variables with highest loadings. We generated for each species all possible models and then ranked these based on the Akaike information criterion for small sample size (AICc) after standardizing the coefficient estimates by their standard deviation. By averaging across all models for each species within ΔAICc < 6 of the best model, we estimated the regression coefficients for those variables that were retained in the averaged model using the R-package “MuMIn”, v. 1.40.4 [63–65]. We considered all models within ΔAICc < 6 of the best model to have similar statistical support [64, 66, 67]. We only considered the estimates of the “full average” as our aim was to determine which factors have the strongest effect on the occurrence of species [68].

To assess whether roost characteristics influence the number of species that can use a specific roost, we fitted a generalized linear regression model in R with the maximum number of bat species observed in a roost as the dependent variable and roost parameters that described the physical characteristics of the roost (fallen vs. standing tree, inside height, diameter above entry, area of entry) as explanatory variables. Due to the skewed distribution of the dependent variable, i.e. counts of species in roosts, we modelled this with a Poisson distribution.

## Supplementary Information

**Additional file 1: Table S1.** Number of roosts and observations of bat species in roosts in different habitats (forest vs. disturbed). **Fig. S1.** GLMM beta parameter estimates for the effect of the presence or abundance of selected other bat species on the incidence of a focal bat species in natural and in artifical roosts in the forest habitat and artificial roosts in the disturbed habitat. **Table S2.** Roost variables used for modelling species-specific roost selection, their mean and range in roosts occupied by each of six focal bat species, and the percentage of roosts occupied by each bat species that were in standing trees. **Fig. S2.** Results of GLM on the effect of roost parameters on the total number of bat species using natural roosts (not necessarily simultaneously). **Fig. S3.** Temperature during five days at three different heights inside a bat roost in a large hollow tree.

## Data Availability

The data supporting the results of this study and the R code are available from the OSF repository: 10.17605/OSF.IO/AKRH9.
