# Supplementary material for: Mixed-species groups in bats: non-random roost associations and roost selection in neotropical understory bats
Source: Front Zool. 2021 Oct 12;18:53. doi: 10.1186/s12983-021-00437-6 (PMC8507185; doi:10.1186/s12983-021-00437-6)
Supplement: Supplementary file 1 — Additional file 1: Table S1. Number of roosts and observations of bat species in roosts in different habitats (forest vs. disturbed). Fig. S1. GLMM beta parameter estimates for the effect of the presence or abundance of selected other bat species on the incidence of a focal bat species in natural and in artifical roosts in the forest habitat and artificial roosts in the disturbed habitat. Table S2. Roost variables used for modelling species-specific roost selection, their mean and range in roosts occupied by each of six focal bat species, and the percentage of roosts occupied by each bat species that were in standing trees. Fig. S2. Results of GLM on the effect of roost parameters on the total number of bat species using natural roosts (not necessarily simultaneously). Fig. S3. Temperature during five days at three different heights inside a bat roost in a large hollow tree. [file 12983_2021_437_MOESM1_ESM.pdf]

Supplementary information – Additional file 1

Mixed-species groups in bats: non-random roost associations and roost selection in Neotropical understory bats

Detlev H. Kelm (☒), Ulf Toelch, Mirkka M. Jones

Table S1: Number of roosts and observations of bat species in roosts in different habitats (forest vs disturbed)

			Natural roost (forest habitat)	Artificial roost (forest habitat)	Artificial roost (disturbed habitat)
Total n roosts / n controls			89 / 520	21 / 585	23 / 658
**Species**	**Diet**	**Abbreviation**	% roost / % controls		
Saccopteryx bilineata	Insects	S.bil	22 / 9	0 / 0	0 / 0
Peropteryx kappleri	Insects	P.kap	6 / 5	0 / 0	0 / 0
Trachops cirrhosis	Insects / vertebrates	T.cir	6 / 3	52 / 11	0 / 0
Micronycteris microtis	Insects	M.mic	25 / 11	62 / 9	35 / 5
M. hirsuta	Insects	M.hir	8 / 2	10 / <1	0 / 0
Glossophaga sp.	Nectar / fruits	G.sp.	30 / 13	76 / 21	87 / 40
G. soricina	Nectar / fruits	G.sor	0 / 0	0 / 0	39 / 2
G. commissarisi	Nectar / fruits	G.com	30 / 13	76 / 21	61 / 5
Hylonycteris underwoodi	Nectar	H.und	8 / 2	0 / 0	0 / 0
Carollia castanea	Fruits	C.cast	26 / 15	10 / <1	4 / <1
C .perspicillata	Fruits	C.per	22 / 13	95 / 24	83 / 14
C. sowelli	Fruits	C.sow	20 / 9	81 / 26	43 / 4
C. perspicillata/sowelli	Fruits	C.per/C.sow	36 / 24	100 / 60	96 / 60
Desmodus rotundus	Blood	D.rot	8 / 12	14 / 1	0 / 0

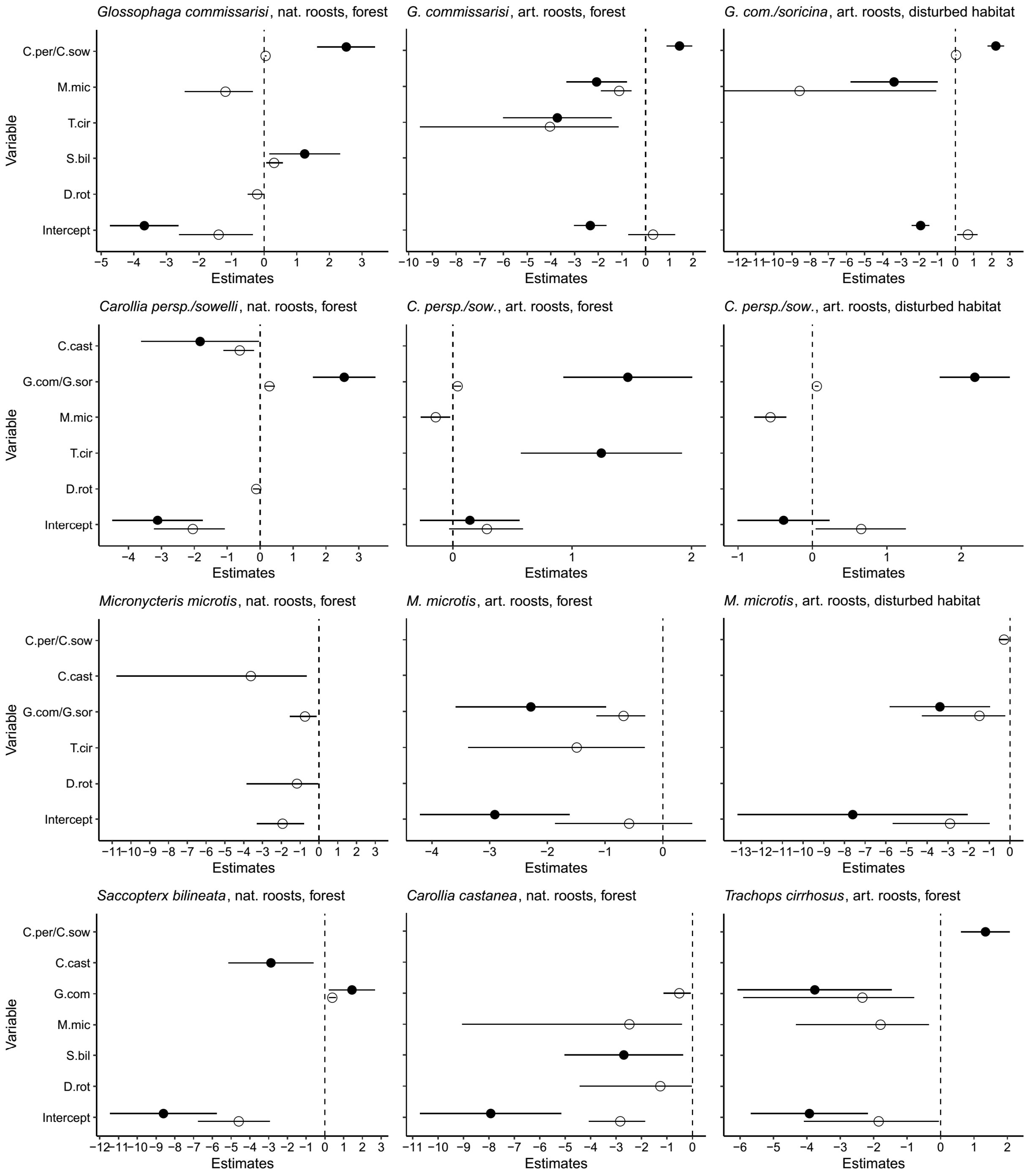

Fig. S1: GLMM beta parameter estimates for the effect of the presence (black circles) or abundance (open circles) of selected other bat species on the incidence of a focal bat species in natural and in artifical roosts in the forest habitat and artificial roosts in the disturbed habitat. The data for the sibling species *Carollia perspicillata* and *C. sowelli* were pooled. The plots only show those species for which a significant positive or negative effect of co-occurrence was detected. Models without any significant effects are not presented. For species abbreviations see the text and table S1. The error bars are 95% credible intervals.

Table S2: Roost variables used for modelling species-specific roost selection, their mean and range (in parentheses) in roosts occupied by each of six focal bat species, and the percentage of roosts occupied by each bat species that were in standing (not fallen) trees. For species abbreviations see the text and table S1.

Species	Roosts (n)	Roost volume (m^3)	Size of the entrance (m^2)	Distance of the roosting site from the entrance (m)	Distance of the roosting site above the ground (m)	Vegetation density (scale 0-10)	Canopy height (m)	Roosts in standing trees (%)
G.com	24	6.7 (0.4-32.6)	0.7 (0.1-3.0)	3.5 (1.0-11.2)	4.8 (0.6-17.0)	4.1 (2-8)	34.8 (20-45)	88
C.per	18	9.6 (1.2-32.6)	0.9 (0.1-3.0)	3.7 (1.0-10.3)	5.7 (2.2-12.3)	4.1 (2-6)	38.6 (25-45)	100
C.sow	18	3.5 (0.4-18.8)	0.4 (0.1-1.5)	3.1 (1.0-10.3)	3.8 (0.6-12.3)	4.3 (2-8)	32.3 (20-45)	78
C.cast	18	1.3 (0.1-4.5)	0.9 (0.1-2.9)	1.2 (0.2-5.7)	2.4 (0.5-6.0)	4.4 (3-6)	30.6 (20-40)	61
M.mic	16	2.9 (0.1-18.8)	0.6 (0.1-5.0)	3.3 (0.5-9.0)	1.1 (0.3-5.0)	4.8 (4-7)	28.1 (15-35)	19
S.bil	11	11.6 (0.2-32.6)	1.3 (0.1-3.0)	2.5 (0.3-4.8)	4.8 (3.0-7.5)	3.9 (2-5)	36.4 (20-45)	100

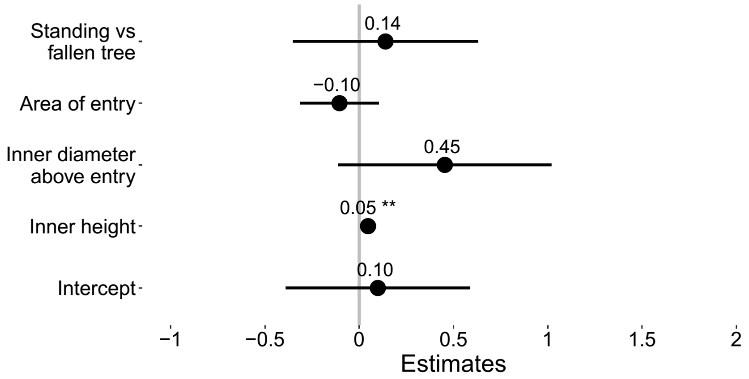

Fig. S2: Results of GLM on the effect of roost parameters on the total number of bat species using natural roosts (not necessarily simultaneously). The numbers next to the points representing beta parameter estimates are p-values and the asterisks indicate a significant effect (** p<0.001)

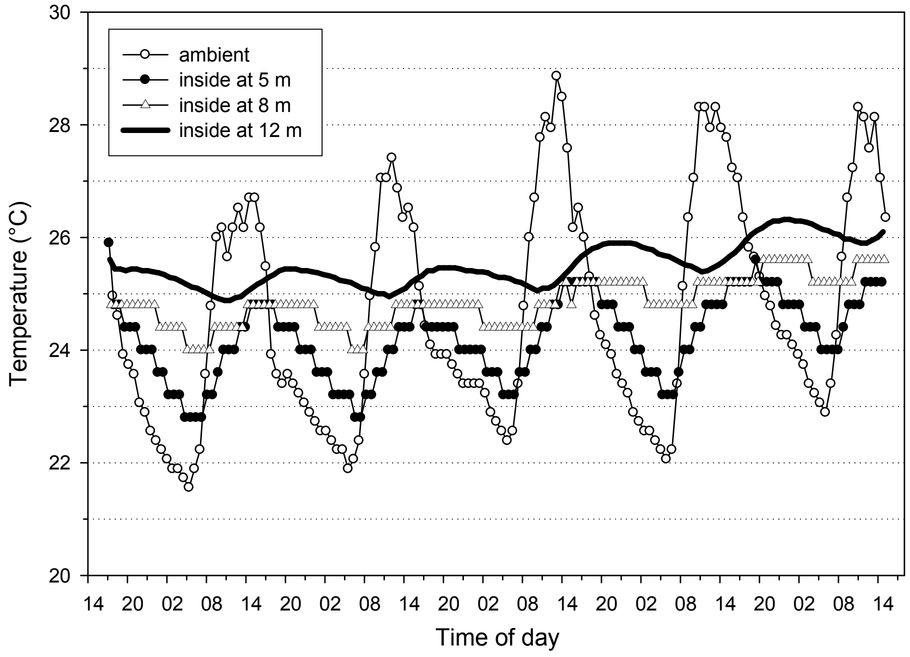

Fig. S3: Temperature during five days at three different heights inside a bat roost in a large hollow tree
